# Complexes With Biologically Active Ligands. Part 4. Coordination Compounds of Chlorothiazide With Transition Metal Ions Behave as Strong Carbonic Anhydrase Inhibitors

**DOI:** 10.1155/MBD.1996.79

**Published:** 1996

**Authors:** Claudiu T. Supuran

**Affiliations:** 1 University of Florence Laboratory of Inorganic and Bioinorganic Chemistry Via Gino Capponi 7 Firenze I-50121 Italy

## Abstract

Complexes of the diuretic benzothiadiazine derivative chlorothiazide (6-chloro-7-sulfamoyl- 1,2,4-benzothiadiazine-1,1-dioxide) with V(IV); Fe(II); Co(II); Ni(II); Cu(II), Ag(I) and U(VI) were prepared and characterized by elemental analysis, spectroscopic, thermogravimetric, magnetic and conductimetric measurements. The complexes behave as effective inhibitors for two isozymes (I and II) of carbonic anhydrase (CA).


### COMPLEXES WITH BIOLOGICALLY ACTIVE LIGANDS. PART 4. COORDINATION COMPOUNDS OF CHLOROTHIAZIDE WITH TRANSITION METAL IONS BEHAVE AS STRONG CARBONIC ANHYDRASE INHIBITORS

Claudiu T. Supuran

University of Florence, Laboratory of Inorganic and Bioinorganic Chemistry,
Via Gino Capponi 7, I-50121, Firenze, Italy

**Abstract**: Complexes of the diuretic benzothiadiazine derivative chlorothiazide (6-chloro-7-sulfamoyl-1,2,4-benzothiadiazine-1,1-dioxide) with V(IV); Fe(II); Co(II); Ni(II); Cu(II), Ag(I) and U(VI) ) were prepared and characterized by elemental analysis, spectroscopic, thermogravimetric, magnetic and conductimetric measurements. The complexes behave as effective inhibitors for two isozymes (I and II) of carbonic anhydrase (CA).

### Introduction

Benzothiadiazines constitute an important class of pharmacological agents[2,3] with wide applications in clinical medicine as diuretics, antihypertensive drugs as well as hyperglycemic agents among others.[3,4] Originally developed as inhibitors of the enzyme carbonic anhydrase (CA, EC 4.2.1.1), these compounds exhibited different pharmacological properties, such as increased elimination of salt in urine, which ultimately led to the development of saluretic drugs such as chlorothiazide, hydrochlorothiazide, cyclothiazide, etc.[2-5] as well as to the high-ceiling diuretics of the furosemide type.[5]

In previous papers[1,6] we investigated the coordination chemistry of diazoxide **1**[6] and chlorothiazide **2**[1] as well as the biological activity (as enzyme inhibitors) of the prepared metal complexes. It was proved that transition metal ions such as Cu(II); Zn(II); Hg(II) or Co(II) among others lead to coordination compounds with greatly increased CA inhibitory properties as compared to the original sulfonamides. Moreover, the complexes of chlorothiazide **2** were more effective as compared to those of diazoxide **1**. As only the chlorothiazide complexes of Zn(II); Cd(II); Hg(II) and of some main group cations were reported,[1] in this paper we extend the study to some other biologically relevant cations.

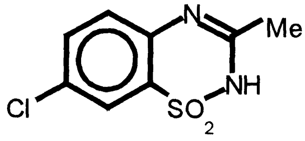

**1 : HDZO**

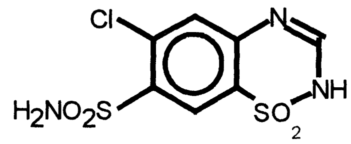

**2: HCTZ**

The present paper describes the preparation of complexes of the conjugated base of **2** with V(IV); Fe(III); Co(II); Ni(II); Cu(II); Ag(I) and U(VI), their characterization as well as biological activity data for the inhibition of two CA isozymes (erythrocyte CA I and II enzymes, which are the predominant and mostly investigated cytoplasmic CAs[5,7]).

### Materials and Methods

IR spectra were obtained in KBr pellets, with a Perkin Elmer 1600 instrument, in the range 200 - 4000 $cm^{-1}$. Electronic spectra were obtained by the diffuse reflectance technique in MgO as reference, with a Perkin Elmer Lambda 17 apparatus. Solution electronic spectra were recorded in acetonitrile with a Cary 3 instrument. Conductimetric measurements were done in DMF solutions, at 25°C (concentrations of 1 mM of complex) with a Fisher conductimeter. Magnetic susceptibility measurements were done at room temperature by Faraday's method, using $CoHg(NCS)_4$ as standard. Elemental analyses were done by combustion for C,H,N with an automated Carlo Erba analyzer, and gravimetrically for the metal ions, and were ± 0.4% of the theoretical values. Thermogravimetric measurements were done in air, at a heating rate of 10°C/min., with a Perkin Elmer 3600 thermobalance.

Chlorothiazide, metal salts (vanadyl sulfate; iron(III) perchlorate; M(II) nitrates, where M = Co, Ni, Cu; silver(I) nitrate and uranyl nitrate) and solvents were from Aldrich or Merck and were used without further purification. Human isozymes CA I and II, buffers and 4-nitrophenyl acetate were from Sigma. Inhibitors were assayed spectrophotometrically at 400 nm, by the method of Pocker and Stone[8] for the inhibition of 4-nitrophenyl acetate hydrolysis catalyzed by the two CA isozymes.

**Synthesis of coordination compounds 4-10**

A *cold* solution of chlorothiazide sodium salt (NaCTZ, **3**) was prepared by suspending HCTZ in the stoichiometric amount of 2N NaOH solution, working at 0-5°C. Mention should be made that the benzothiadiazinic ring is not very stable in the presence of bases, being cleaved to orthanilamide derivatives.[9] Still, at room temperature and in 2N NaOH, this ring is cleaved in about 150 hours,[9] so that, presumably, no decomposition occurred during the experiments reported here, in which complexes were prepared in about 0.5 - 1 hours. The cold solution obtained above, was mixed with a methanolic-aqueous solution of metal salts in molar ratios of 2:1(for the divalent cations, the vanadyl and uranyl salts); 1:1 (for Ag(I)) or 3:1 (for Fe(III)). The obtained reaction mixture was stirred magnetically at room temperature for 0.5 - 1 hours. The complexes so obtained were collected by filtration and air-dried. Due to their poor solubility in the majority of solvents (excepting for DMSO and DMF) recrystallization attempts of the new complexes were unsuccessful.

**Results and Discussion**

The chlorothiazide complexes **4-10** prepared in the present work together with their elemental analysis (within ± 0.4% of the theoretical values calculated for the proposed formulas) and TG data are shown in Table I.

Table I: The prepared chlorothiazide complexes **4-10**, their elemental analysis and TG data (CTZ stands for the endocyclic $SO_2NH$ deprotonated species of chlorothiazide).

| No. | Compound | Analysis (calc./found) | | | | |
|---|---|---|---|---|---|---|
| | | %M[a] | %C[b] | %H[b] | %N[b] | $\%H_2O$[c] |
| **4** | $[VO(CTZ)_2(OH_2)_2]$ | 7.0/6.8 | 26.6/26.8 | 2.5/2.4 | 11.6/11.5 | 5.0/5.2[d] |
| **5** | $[Fe(CTZ)_3(OH_2)_3]$ | 5.3/5.3 | 27.8/27.7 | 2.6/2.3 | 12.1/12.0 | 5.2/5.0[e] |
| **6** | $[Co(CTZ)_2(OH_2)_4]$ | 7.8/7.9 | 25.6/25.6 | 2.9/2.6 | 11.2/11.4 | 9.6/9.5[f] |
| **7** | $[Ni(CTZ)_2(OH_2)_4]]$ | 7.8/7.6 | 25.7/25.5 | 2.9/3.0 | 11.2/11.0 | 9.6/9.7[f] |
| **8** | $[Cu(CTZ)_2(OH_2)_4]$ | 8.4/8.3 | 25.5/25.2 | 2.9/2.5 | 11.1/11.1 | 9.5/9.3[f] |
| **9** | $[Ag(CTZ)(OH_2)]$ | 24.8/25.0 | 22.1/22.2 | 2.0/1.8 | 9.6/9.4 | 4.1/3.8[g] |
| **10** | $[UO_2(CTZ)_2(OH_2)_3]$ | 25.3/25.5 | 20.4/20.1 | 2.1/2.1 | 8.9/8.5 | 5.7/5.5[e] |

[a]By gravimetry; [b]By combustion; [c]By TG analysis; [d] Corresponding to 2 $H_2O$, lost between 150-175°C; [e] Corresponding to 3 $H_2O$, lost between 150-180°C; [f] Corresponding to 4 $H_2O$, lost between 140-180°C; [g] Corresponding to 1 $H_2O$, lost between 160-185°C.

The new complexes were also characterized by spectroscopic, magnetic and conductimetric data (Tables II and III).

In the IR spectra of complexes **4-10**, the only major changes as compared to the spectrum of **2**, concern the sulfonamido vibrations, in the region 1100-1400 $cm^{-1}$. As shown in the previous paper,[1] due to the fact that chlorothiazide has two types of sulfonamido moieties (the endo- and the exocyclic ones), two bands appear for both the symmetrical as well as antisymmetrical $SO_2$ vibrations (Table II). As in the previously reported complexes,[1] only the band corresponding to the endocyclic $SO_2$ group undergoes shifts towards lower wavenumbers (with 27-35 $cm^{-1}$ for the symmetrical vibrations, and 20-36 $cm^{-1}$, for the antisymmetrical ones, respectively) in complexes **4-10**, proving the participation of the endocyclic $SO_2N$ moiety in interaction with the metal ions, as well as the fact that the 7-substituent, the $SO_2NH_2$ moiety is not involved in complexation. Another difference in the IR spectra of the complexes consists in the presence of vibrations under 400 $cm^{-1}$, tentatively assigned as due to M-N and M-O vibrations. [1,7] One reaches the same conclusion regarding the donor atom of chlorothiazide in its complexes reported here, by comparing solution electronic spectra of **2**, its sodium salt **3** and complexes **4-10**. In the last compounds as well as in the sodium salt, only one broad band appears at 323 nm, whereas in **2** two absorption maxima were evidenced. This type of modification of electronic spectrum in the presence of bases or by formation of complexes was previously documented for related systems (heterocyclic sulfonamides [1,7,10] or for the related benzothiadiazine derivative, hydrochlorothiazide[11]). Thus, in all these complexes, as in those previously reported,[1] the chlorothiazidate anion behaves as a monodentate ligand, interacting with the metal ions through the N-2 atom.

Table II: IR and solution electronic spectral data for compounds **2-10**.

| **Comp.** | **Color** | **IR. Spectra** [a] ,$cm^{-1}$ | | | **UV Spectra** [b] |
|---|---|---|---|---|---|
| | | $\nu$(M-L) | $\nu(SO_2)^s$ | $\nu(SO_2)^{as}$ | $\lambda_{max}$, nm (lgε) |
| **2** | - | - | 1117, 1180 | 1325, 1377 | 278 (3.82); 328 (2.13) |
| **3** | - | - | 1121, 1185 | 1345 | 323 (4.18) |
| **4** | gray | 345;398 | 1085, 1180 | 1300, 1375 | 323 (4.20) |
| **5** | brown | 340;398 | 1086, 1180 | 1302, 1377 | 322 (4.73) |
| **6** | pink | 350;400 | 1090, 1180 | 1300, 1377 | 323 (4.19) |
| **7** | green | 348;400 | 1087, 1180 | 1300, 1376 | 323 (4.25) |
| **8** | emerald | 350;400 | 1087, 1179 | 1303, 1375 | 323 (4.22) |
| **9** | white | 337;396 | 1082, 1180 | 1305, 1377 | 323 (3.98) |
| **10** | yellow | 330;398 | 1084, 1179 | 1289, 1377 | 323 (4.27) |

[a] In KBr; [b] In acetonitrile.

Supplementary information regarding the stereochemistry of metal ions in the prepared complexes was obtained by means of diffuse reflectance electronic spectroscopy as well magnetic moment measurements (Table III).

Table III: Diffuse reflectance spectra, magnetic moments and proposed geometries for complexes **4-10**.

| **Complex** | **Electronic spectra** ($\nu$, $cm^{-1}$)[a] | $\mu_{eff}$ (BM)[b] | **Geometry** |
|---|---|---|---|
| **4** | 25,900; 15,500; 11,900(sh) | 1.85 | square pyramidal |
| **5** | 24,650; 20,300; 10,500 | 5.75 | octahedral |
| **6** | 25,600; 18,700(sh); 15,600 | 5.25 | octahedral |
| **7** | 17,000; 12,500 | 3.46 | octahedral |
| **8** | 16,200 | 1.90 | octahedral |
| **9** | c | d | linear |
| **10** | 23,400, 21,600 | 0.67 | pentagonal bipyramidal |

[a] In MgO as standard material; [b] At room temperature; [c] No transitions in this spectral region seen; [d] Diamagnetic.

From the above data, it is seen that the V(IV) derivative **4** is in its predilect geometry - square pyramidal,[12] similarly with the related diazoxide complex previously reported,[6] whereas the iron derivative **5** in octahedral geometry (electronic spectrum and magnetic moment typical for this geometry of Fe(III)[13]). In these derivatives the water molecules, coordinated to the metal ions as proved by TG data (Table I) complete the coordination sphere around the metal, in addition to the two and three, respectively, chlorothiazidate ligands.

The other metal ions showing an interesting RD spectrum in their complexes with chlorothiazide are Co(II) in **6**, Ni(II) in **7** and Cu(II) in **8**. Two bands were detected in the RD spectrum of **6**, situated at 25,600 and 15,600 $cm^{-1}$ , respectively, assigned as the $\nu_3$ and $\nu_2$ transitions, as well as a shoulder at 18,700 $cm^{-1}$. The $\nu_1$ calculated from the Lever tables is 7,260 ,which leads to a $\nu_2/\nu_1$ ratio in the range of 2.1-2.2, which, correlated with a magnetic moment of 5.25 BM at room temperature, suggests an octahedral geometry for the Co(II) ion.[14] This is also supported by TG analysis data (Table I), which proved that the four water molecules coordinated to the metal ion are lost in a single step, between 140-180 °C. For the Ni(II) complex 7, two weak transitions were evidenced in the RD spectrum, at 17,000 and 12,500 $cm^{-1}$ attributed to the $\nu_1$ and $\nu_2$ transitions of Ni(II) in octahedral surrounding.[5,6] This is also supported by the magnetic moment of 3.46 BM.[6,15] The Cu(II) complex **8** shows a large structureless band centered at 16,200 $cm^{-1}$ and a magnetic moment of 1.90 BM, indicating probably a distorted octahedral geometry of Cu(II).[6,16] In the last two complexes water is also directly bound to the metal ions, since by means of TG analysis it was shown that this is lost in one step, between 140-180°C. The Ag(I) derivative **9** probably possesses a linear geometry with the chlorothiazidate and water ligands occupying the two coordination sites of silver.[17] Finally, the electronic spectrum and the rest of analytical data of the uranyl derivative **10** are consistent with heptacoordinated U(VI), similarly to other such derivatives previously reported by us.[18] Conductimetric measurements (data not shown) proved all complexes **4-10** to be of the non-electrolyte type, in contrast to the sodium salt **3** which behaved as an 1:1 electrolyte. All these data are consistent with the structures proposed above for the new complexes of chlorothiazide.

Inhibition data against the major red cell CA isozymes (CA I and CA II) with the new complexes are shown in Table IV.

Table IV : CA I and II inhibition data for 4-nitrophenyl acetate hydrolysis reaction,[8] in the presence of compounds **1-10.**

| Inhibitor | $IC_{50}$ ($\mu$M)[a] | |
|---|---|---|
| | CA I | CA II |
| **1 (HDZO)** | 115 | 58 |
| **2 (HCTZ)** | 104 | 20 |
| **4** | 62 | 14 |
| **5** | 50 | 13 |
| **6** | 37 | 10 |
| **7** | 44 | 8 |
| **8** | 6 | 1.2 |
| **9** | 14 | 2.4 |
| **10** | 13 | 1.5 |

[a] Molarity of inhibitor producing a 50% decrease of enzyme specific activity for the esterasic activity of these enzymes.

As seen from the above data, all the new complexes are stronger inhibitors, towards both isozymes, as compared to chlorothiazide or the related benzothiadiazine, diazoxide. Cations leading to very effective inhibitors were Cu(II); Ag(I) and U(VI). In fact, all these three cations were previously reported to form complexes with aromatic/heterocyclic sulfonamides showing 100-1000 times better inhibition profiles, as compared to the parent ligands.[7,10,13] Although in previous papers we explained this phenomenon as due to binding of the cations contained in the complex inhibitors to residue His-64 in CA II active site, it seems that supplementary binding sites are available at the entrance of CA II active site,[1] as we[19] recently discovered a histidine cluster in that region, formed from residues 3, 4, 10, 15, 17 and 64. This would explain the higher affinity of coordination compounds to this isozyme, as well as the catalytic properties of the respective isozyme *per se*. In principle, by using this approach for the design of novel CA inhibitors, it would be possible to obtain compounds with specificity towards certain isozymes and their selective inhibition. This would eventually allow us to understand the physiological function for some of the eight CA isozymes, as presently this seem to be certain only for CA II and CA IV.[7]

### References

1. C.T.Supuran, *Main Group Met. Chem., in press.*
2. F.C.Novello and J.M.Sprague, *J.Am.Chem.Soc.*, **1957**, *79*, 2028-2029.
3. a) K.H.Beyer and J.E.Baer, *Pharmacol.Rev.*, **1961**, *13*, 517-562; b) I.M.Weiner, in "The Pharmacological Basis of Therapeutics", 8th Edition, A.G.Gilman, T.W.Rall, A.S.Nies and P.Taylor Eds., Pergamon Press, New York, 1990, pp. 713-732.
4. a) M.B.Standen, J.M.Quayle, N.W.Davies, J.E.Brayden, Y.Huang and M.T.Nelson, *Science*, **1989**, *245*, 177-180; b) J.G.Gerben and A.S.Nies, in "The Pharmacological Basis of Therapeutics", 8th Edition, A.G.Gilman, T.W.Rall, A.S.Nies and P.Taylor Eds., Pergamon Press, New York, 1990, pp. 784-813; c) S.R.Levin, M.A.Charles, M.O'Connor and G.M.Grodsky, *Am.J.Physiol.*,**1975**, *299*, 49-54; d) C.R.Kahn and Y.Schechter, in "The Pharmacological Basis of Therapeutics", 8th Edition, A.G.Gilman, T.W.Rall, A.S.Nies and P.Taylor Eds., Pergamon Press, New York, 1990, pp. 1463-1495.
5. T.H.Maren, *Pharmacol.Rev.*, **1967**, *47*, 595-782.
6. C.T.Supuran, *Metal Based Drugs*, **1995**, 2, 327-330.
7. C.T.Supuran, in "Carbonic Anhydrase and Modulation of Physiologic and Pathologic Processes in the Organism", I.Puscas Ed., Helicon, Timisoara 1994, pp. 29-111.
8. Y.Pocker and J.T.Stone, *Biochemistry*, **1967**, *6*, 668-679.
9. a) J.H.Freeman and E.C.Wagner, *J.Org.Chem.*, **1951**, *16*, 815-837; b) F.C.Novello, S.C.Bell, E.L.A.Abrams, C.Ziegler and J.M.Sprague, *J.Org.Chem.*, **1960**, *25*, 970-981; c) J.K.Wales, S.V.Krees, A.M.Grant, J.K.Viktora and F.W.Wolff, *J.Pharmacol.Exp.Ther.*, **1968**, *164*, 421-431.
10. G.Alzuet, S.Ferrer, J.Borras and C.T.Supuran, *Roum.Chem.Quart.Rev.*, **1994**, *2*, 283-300.
11. H.Abdine, M.A.Elsayed and Y.M.Elsayed, *J.Assoc.Off.Anal.Chem.*, **1978**, *61*, 695-701.
12. C.J.Balhausen and H.Gray, *Inorg.Chem.*, **1962**, *1*, 111-115.
13. C.T.Supuran, *Rev. Roum.Chim.*, **1992**, *37*, 849-855.
14. L.Banci, A.Bencini, C.Benelli, D.Gatteschi and C.Zanchini, *Struct.Bonding*, **1982**, *52*, 37-79.
15. B.N.Figgis, "Introduction to Ligand Field Theory", J.Wiley &Sons, New York, 1966.
16. G. Alzuet, J.Casanova, J.Borras, J.A.Ramirez and O.Carugo, *J.Inorg.Biochem.*, **1995**, *57*, 219-234
17. A.Bult, in "Metal Ions in Biological Systems", H.Sigel Ed., Vol. 16, , M.Dekker, New York, 1983, pp. 261-278
18. C.T.Supuran, *Rev.Roum.Chim.*, **1993**, *38*, 229-236.
19. I.Bertini, F.Briganti, A.Scozzafava and C.T.Supuran, *manuscript in preparation.*

**Received: January 15, 1996 - Accepted: February 26, 1996 - Received in revised camera-ready format: March 11, 1996**

